# Head-to-Head in Heart Failure: Comparative Insights on Empagliflozin and Dapagliflozin

**DOI:** 10.3390/biomedicines13102422

**Published:** 2025-10-03

**Authors:** Dragos Cozma, Cristina Văcărescu, Claudiu Stoicescu

**Affiliations:** 1 Institute of Cardiovascular Diseases Timisoara, 300310 Timisoara, Romania; 2 Cardiology Department, “Victor Babeș” University of Medicine and Pharmacy, 300041 Timisoara, Romania; 3 Research Center of the Institute of Cardiovascular Diseases Timisoara, 300310 Timisoara, Romania; 4 Cardiology and Cardiovascular Surgery Department, University of Medicine and Pharmacy Carol Davila, 030167 Bucharest, Romania; claudiu.stoicescu@umfcd.ro; 5 Cardiology and Cardiovascular Surgery Department, University Emergency Hospital, 050098 Bucharest, Romania

**Keywords:** heart failure therapy, SGLT2 inhibitors advancements, empagliflozin vs. dapagliflozin, cardiorenal protection, beyond glycemic control

## Abstract

Heart failure (HF) remains a leading cause of morbidity and mortality globally, with increasing prevalence driven by aging populations and comorbidities such as diabetes mellitus. Recent advances have highlighted sodium-glucose cotransporter-2 (SGLT2) inhibitors, particularly empagliflozin and dapagliflozin, as effective agents in HF management across a broad spectrum of ejection fractions. Initially developed for glycemic control in type 2 diabetes, both drugs have demonstrated significant cardiovascular benefits, including reductions in HF hospitalizations and improvements in symptoms and quality of life. Their mechanisms extend beyond glucose lowering, involving natriuresis, osmotic diuresis, improved myocardial energetics, reduced sympathetic activation, and anti-inflammatory effects. While empagliflozin and dapagliflozin share a core renal mechanism via selective SGLT2 inhibition, subtle differences in pharmacokinetics, potency, and tissue selectivity may influence their clinical profiles. Emerging evidence suggests empagliflozin may confer stronger benefits in heart failure with reduced ejection fraction (HFrEF), while dapagliflozin could offer enhanced efficacy in heart failure with preserved ejection franction (HFpEF), although head-to-head comparisons are lacking. This review synthesizes current evidence comparing the mechanisms of action and clinical performance of empagliflozin and dapagliflozin in HF, providing insight into agent selection and future directions in therapy personalization.

## 1. Introduction

Heart failure (HF) is a complex clinical syndrome that affects millions of individuals worldwide, with a rising prevalence due to aging populations and increasing comorbidities such as diabetes mellitus. The pathophysiology of HF involves a range of systemic abnormalities, including neurohormonal activation and inflammation, impacting both inotropic and/or lusitropic heart function and contributing to significant morbidity and mortality [1].

In recent years, sodium-glucose cotransporter-2 (SGLT2) inhibitors, particularly empagliflozin and dapagliflozin, have emerged as promising therapeutic agents in the management of HF. Both agents have demonstrated beneficial effects on clinical outcomes in heart failure, both in patients with reduced ejection fraction (HFrEF) and preserved ejection fraction (HFpEF) [2,3,4,5].

This review updates the current understanding and comparative effectiveness of empagliflozin and dapagliflozin in heart failure, exploring their clinical efficacy, safety profiles, mechanistic insights, and the latest data from key trials.

To ensure a comprehensive and up-to-date synthesis of the evidence, we performed a narrative review of the literature using the PubMed, Scopus, and Web of Science databases. The search included articles published between January 2010 and August 2025. We used a combination of Medical Subject Headings (MeSH) terms and keywords, including “SGLT2 inhibitors,” “empagliflozin,” “dapagliflozin,” “heart failure,” “cardiovascular outcomes,” and “combination therapy.” Both randomized controlled trials and high-quality observational studies were included, with a focus on human studies published in English. Additional references were identified through manual screening of bibliographies from key articles and recent guidelines. Priority was given to high-impact studies, recent clinical trials, and meta-analyses directly relevant to the scope of this review.

## 2. Mechanism of Action

Empagliflozin and dapagliflozin are members of the sodium-glucose cotransporter 2 (SGLT2) inhibitor class, which exert their primary pharmacological action by inhibiting SGLT2 in the proximal renal tubule. SGLT2 is responsible for reabsorbing approximately 90% of filtered glucose from the glomerular filtrate. By blocking this transporter, both agents reduce glucose and sodium reabsorption, resulting in increased urinary glucose excretion (glucosuria) and natriuresis [6,7]. This mechanism underpins their glucose-lowering efficacy in patients with type 2 diabetes mellitus.

Beyond glycemic control, SGLT2 inhibitors have demonstrated significant cardiovascular benefits, particularly in patients with heart failure [8]. The following pleiotropic effects contribute to their cardioprotective profile [9]:

- Osmotic diuresis and natriuresis: By inhibiting sodium and glucose reabsorption, these agents promote mild diuresis and natriuresis, leading to reduced plasma volume. This volume reduction decreases cardiac preload and afterload, thereby alleviating hemodynamic stress on the failing heart.
- Reduction in cardiac remodeling: Preclinical and clinical studies suggest that SGLT2 inhibitors may exert anti-inflammatory and antifibrotic effects, contributing to attenuation of maladaptive myocardial remodeling.
- Improved endothelial function and vascular tone: These drugs may enhance endothelial function and lower systemic vascular resistance, thereby improving vascular health.
- Sympathetic nervous system modulation: SGLT2 inhibition has been associated with reduced sympathetic nervous system activation, a key pathophysiological driver of heart failure progression [9].

While empagliflozin and dapagliflozin share a common mechanism through SGLT2 inhibition, they may differ slightly in their pharmacokinetic and pharmacodynamic profiles. However, their core renal and cardiovascular actions are fundamentally similar, with both demonstrating efficacy in glycemic control and heart failure management across diverse patient populations At a mechanistic level, SGLT2 inhibitors exert multi-organ effects beyond glycemic control. Their inhibition of renal glucose and sodium reabsorption induces natriuresis and a reduction in intravascular volume without triggering neurohormonal activation. Importantly, emerging data suggest that SGLT2 inhibitors reduce myocardial inflammation and fibrosis via suppression of proinflammatory cytokines (e.g., TNF-α, IL-6), inhibition of the myocardial sodium-hydrogen exchanger (NHE1), and modulation of ketone metabolism and mitochondrial energetics. These actions may contribute to improved diastolic function, enhanced myocardial efficiency, and attenuation of maladaptive remodeling. When combined with angiotensin receptor-neprylisin inhibitors (ARNIs) or mineralocorticoid receptor antagonists (MRAs), which target neurohormonal and fibrotic pathways through distinct mechanisms, SGLT2 inhibitors may offer a broader and more effective disease-modifying impact. This mechanistic complementarity underscores the rationale for early and concurrent initiation of these agents in patients across the heart failure spectrum [10,11].

Recent advances in molecular and cellular research have significantly expanded our understanding of the pleiotropic effects of SGLT2 inhibitors beyond glycemic control. Notably, SGLT2i therapy has been shown to induce a favorable shift in myocardial substrate utilization, enhancing ketone body oxidation and thereby improving cardiac metabolic efficiency in the failing heart. Moreover, inhibition of the myocardial sodium–hydrogen exchanger (NHE1) reduces intracellular sodium and calcium overload, contributing to improved ventricular relaxation and contractility [11].

Emerging evidence also suggests that SGLT2 inhibitors exert beneficial effects on mitochondrial function, including enhanced biogenesis, improved ATP production, and reduced oxidative stress, all of which may contribute to myocardial protection. Recent work provides compelling data linking SGLT2 inhibition to improved cardiomyocyte calcium handling and mitochondrial respiration, mediated via activation of the AMPK–SIRT1–PGC-1α signaling pathway. These findings underscore the potential of SGLT2 inhibitors to modulate critical intracellular pathways involved in myocardial energetics and remodeling, offering mechanistic support for their clinical efficacy across diverse heart failure phenotypes [12].

Though both drugs share this common mechanism, there are some nuances in their potency and selectivity, pharmacokinetics, and potential for extra-renal effects that may differentiate their impact in clinical settings.

- Potency and Selectivity

**Empagliflozin:** Empagliflozin is a highly selective SGLT2 inhibitor, with a very low affinity for SGLT1 (a transporter primarily found in the intestines). It has greater potency for inhibiting SGLT2 in the kidneys compared to dapagliflozin, which may lead to a more pronounced reduction in renal glucose reabsorption and enhanced diuresis. This could result in more significant improvements in blood pressure and fluid retention in heart failure patients, although this difference may not always be clinically significant across most populations [13,14].

**Dapagliflozin:** While dapagliflozin is also highly selective for SGLT2, it has a slightly lower potency compared to empagliflozin in terms of inhibiting glucose reabsorption in the kidneys. This difference could translate to a slightly weaker effect on urinary glucose excretion. However, it has similar efficacy in managing heart failure, as observed in clinical trials. Dapagliflozin is also considered to have a slightly broader effect across different populations, especially in patients with heart failure with preserved ejection fraction HFpEF [15].

b. Pharmacokinetics (Absorption and Half-Life)

**Empagliflozin:** Empagliflozin has a longer half-life (around 12 h) compared to dapagliflozin, which means it stays in the body longer. This long half-life allows for once-daily dosing and may offer more consistent effects throughout the day. Its absorption is not significantly affected by food intake, ensuring reliable bioavailability [16].

**Dapagliflozin:** Dapagliflozin also has a long half-life of around 10–12 h, which similarly allows for once-daily dosing. Its pharmacokinetic properties are slightly more influenced by food compared to empagliflozin, though this difference is not typically clinically significant for most patients [17].

c. Effects Beyond the Kidneys

Both empagliflozin and dapagliflozin have been shown to exert extra-renal effects that contribute to their beneficial effects in heart failure, but some differences in these effects are hypothesized:

**Empagliflozin:** In addition to its renal effects, empagliflozin may have a stronger impact on myocardial tissue through its potential for reducing myocardial inflammation, fibrosis, and oxidative stress. These effects may contribute to improvements in functional capacity and quality of life in heart failure patients. There is evidence suggesting empagliflozin may have greater efficacy in improving symptoms and quality of life in HFrEF patients, as observed in clinical trials [18,19,20].

**Dapagliflozin:** Dapagliflozin may have a more prominent effect on improving cardiovascular outcomes in heart failure with preserved ejection fraction (HFpEF), possibly through mechanisms beyond its renal and diuretic effects. For instance, dapagliflozin has been suggested to improve vascular stiffness, a key contributor to HFpEF, by reducing vascular inflammation and hypertrophy of the heart [5,21].

Many mechanistic claims remain exploratory or are based on preclinical or secondary analyses, not definitive head-to-head clinical trial comparisons. Empagliflozin and dapagliflozin have shown largely overlapping benefits in both HFrEF and HFpEF, though trial populations and endpoints vary, which might create the appearance of drug-specific advantages. Table 1 provides a structured comparison of the distinct mechanisms of action of empagliflozin and dapagliflozin, highlighting their pharmacodynamic profiles, tissue selectivity, and potential differential effects on cardiovascular, renal, and metabolic pathways. This summary aims to elucidate how subtle pharmacologic variations between the two SGLT2 inhibitors may translate into clinically relevant outcomes.

These nuanced differences should be considered when tailoring HF therapy, especially in regard to heart failure phenotype (HFrEF vs. HFpEF), comorbidities, and the desired outcomes for the patient.

Emerging research is beginning to explore pharmacogenomic and biomarker-based predictors of response to SGLT2 inhibitors in heart failure. Although no pharmacogenomic variant has yet been validated for routine clinical use, preliminary data suggest that polymorphisms affecting *SLC5A2* (the gene encoding SGLT2) and genes related to renal sodium handling may modulate therapeutic efficacy or tolerability. In parallel, biomarkers such as N-terminal pro-B-type natriuretic peptide (NT-proBNP), urinary albumin-to-creatinine ratio, fibrosis markers (e.g., galectin-3), and measures of myocardial strain have shown promise in identifying patients most likely to benefit from SGLT2 inhibition [22]. Subgroup analyses from trials like DELIVER and EMPEROR-Preserved further suggest that patients with elevated baseline natriuretic peptides, mildly reduced EF, or cardiorenal syndrome derive particularly robust benefit. As precision medicine advances, integrating genetic and biomarker profiles may enable more individualized SGLT2i therapy, optimizing outcomes while minimizing adverse effects.

## 3. Evidence in Chronic Heart Failure

### 3.1. Comparison of Empagliflozin and Dapagliflozin in HFrEF

Both empagliflozin and dapagliflozin have demonstrated significant benefits in the treatment of both HFrEF and HFpEF. However, there are several key differences and similarities worth noting.

Despite enrolling similar populations with chronic heart failure and reduced ejection fraction (LVEF ≤ 40%), the EMPEROR-Reduced (*n* =3730) and DAPA-HF (*n* = 4744) trials exhibited key differences in their inclusion criteria and baseline characteristics. Notably, EMPEROR-Reduced applied a stratified NT-proBNP threshold based on baseline LVEF, intentionally selecting patients with more advanced disease. This approach is reflected in the trial’s baseline data, with participants demonstrating a lower mean LVEF (27% vs. 31%) and higher median NT-proBNP levels (≈1900 pg/mL vs. ≈1430 pg/mL) compared to those in DAPA-HF [2,3].

Paradoxically, a smaller proportion of participants in EMPEROR-Reduced had a history of recent heart failure hospitalization (≈31% vs. 47%), and more were classified as NYHA functional class II, suggesting a somewhat less symptomatic cohort despite worse biomarker and functional parameters. In terms of renal function, EMPEROR-Reduced patients also had a lower median estimated glomerular filtration rate (≈61 vs. 66 mL/min/1.73 m^2^), and a higher proportion had estimated glomerular filtration rate (eGFR) ≤ 60 mL/min/1.73 m^2^, indicating greater renal impairment [22].

Both trials maintained high standards of background heart failure therapy, including widespread use of β-blockers, renin–angiotensin–aldosterone system inhibitors, and mineralocorticoid receptor antagonists. However, the use of sacubitril/valsartan was approximately twice as frequent in EMPEROR-Reduced compared to DAPA-HF, although overall uptake remained modest. Importantly, both studies enrolled similar proportions of patients with and without type 2 diabetes mellitus (approximately 50%), supporting the generalizability of findings across glycemic subgroups [2,3,23].

Despite differences in patient populations, both EMPEROR-Reduced and DAPA-HF showed a consistent 25% relative reduction in cardiovascular death or heart failure hospitalization with SGLT2 inhibitors. However, event rates were higher in EMPEROR-Reduced, and only DAPA-HF showed significant reductions in cardiovascular and all-cause mortality. Differences in cardiovascular death between DAPA-HF and EMPEROR-Reduced likely reflect variations in statistical power, follow-up duration, and trial design. Impact on cardiovascular death may be related to greater use of advanced heart failure therapies and a less symptomatic population [2,3].

Statistically, a significant reduction in CV death—within a composite endpoint trial—could be a clinically powerful finding. It supports a mortality benefit, increases confidence in efficacy, and helps differentiate treatments beyond symptom control, but with the limitation given by type of population selected in both trials.

### 3.2. Comparison of Empagliflozin and Dapagliflozin in HFpEF and HFmrEF

The EMPEROR-Preserved and DELIVER trials represent landmark investigations into the use of SGLT2 inhibitors in patients with heart failure with preserved ejection fraction (HFpEF). Despite evaluating different agents—empagliflozin in EMPEROR-Preserved and dapagliflozin in DELIVER—both trials yielded concordant evidence supporting the efficacy of SGLT2 inhibition in this historically treatment-resistant population. However, there are important differences in trial design, patient populations, and inclusion criteria that merit closer examination [5,24].

A primary distinction lies in the inclusion criteria and patient characteristics. EMPEROR-Preserved enrolled patients with left ventricular ejection fraction (LVEF) > 40%, excluding those with mid-range ejection fractions at the lower boundary. DELIVER also used a cutoff of LVEF > 40%, but uniquely included patients with a history of reduced LVEF that had subsequently improved (HFimpEF), reflecting a broader and more contemporary clinical population. Moreover, DELIVER allowed for the enrollment of patients with recently worsened heart failure, including those recently hospitalized or requiring intravenous diuretics, whereas EMPEROR-Preserved excluded patients with hospitalization for heart failure within the prior 90 days. This design likely resulted in a DELIVER cohort with a higher baseline event risk [5,24].

Both trials utilized similar primary composite endpoints of cardiovascular death or heart failure hospitalization (HHF) and demonstrated statistically significant reductions with SGLT2 inhibitor therapy. EMPEROR-Preserved reported a 21% relative risk reduction in the primary endpoint (hazard ration HR 0.79; *p* < 0.001), while DELIVER demonstrated a 18% relative risk reduction (HR 0.82; *p* < 0.001). In both studies, the observed benefit was predominantly driven by reductions in HHF, with no statistically significant difference in cardiovascular mortality. Notably, DELIVER reported a nominal reduction in all-cause mortality (HR 0.94; 95% confidence interval CI, 0.86–1.03; *p* = 0.03), although this was considered exploratory and not adjusted for multiplicity [5,24].

Renal function criteria also differed slightly, with EMPEROR-Preserved including patients with an estimated glomerular filtration rate (eGFR) ≥20 mL/min/1.73 m^2^, whereas DELIVER used a slightly higher cutoff of ≥25 mL/min/1.73 m^2^. The use of natriuretic peptide thresholds for inclusion was broadly similar, though both trials stratified thresholds based on the presence of atrial fibrillation [5,24].

In terms of population size and follow-up, both trials were robust. EMPEROR-Preserved enrolled 5988 patients with a median follow-up of 26.2 months, while DELIVER included 6263 patients with a median follow-up of 2.3 years, further enhancing statistical power [5,24].

In summary, while EMPEROR-Preserved and DELIVER reached qualitatively similar conclusions regarding the role of SGLT2 inhibitors in HFpEF, their methodological and population differences—particularly related to LVEF spectrum, symptom severity, and inclusion of recently decompensated patients—are essential when interpreting trial applicability. Together, the trials confirm a class effect of SGLT2 inhibition in reducing heart failure hospitalizations across the left ventricle ejection fraction (LVEF) continuum, though modest or non-significant effects on mortality persist, emphasizing the need for ongoing mechanistic and comparative studies.

HFmrEF is generally defined as left ventricular ejection fraction (LVEF) ≈ 41–49% (definitions vary slightly between guidelines and trials). Because patients in this band share pathophysiologic features of both HFrEF and HFpEF, prespecified and post hoc subgroup analyses from the large SGLT2 inhibitor outcome trials provide the best evidence about efficacy in HFmrEF.

Both empagliflozin and dapagliflozin trials prospectively enrolled patients with LVEF above the classic HFrEF threshold and therefore provide reasonably large HFmrEF subgroups. In EMPEROR-Preserved and DELIVER, treatment reduced the composite of cardiovascular death or worsening heart failure across the LVEF spectrum, with effect sizes that were generally strongest at lower EF ranges and attenuated at higher EF. EMPEROR-Preserved demonstrated a significant relative risk reduction in the primary composite endpoint overall and showed benefit in lower EF strata; prespecified and subsequent subgroup analyses report consistent reductions in HF events for patients with LVEF in the 41–49% range [25]. Likewise, DELIVER’s prespecified analyses found reductions in total heart-failure events and cardiovascular death for patients with mildly reduced EF (the trial included a substantial HFmrEF cohort) [26].

For patients with HFmrEF, current randomized evidence supports using an SGLT2 inhibitor (either empagliflozin or dapagliflozin) to reduce the risk of worsening HF and to improve symptoms/quality of life. Choice between agents should be guided by overall patient comorbidities, renal function, formulary/access, and clinician experience rather than expectation of a large differential efficacy signal in the HFmrEF band, because randomized trials show broadly consistent benefit in this EF range [27].

### 3.3. Comparative Meta-Analyses and Head-to-Head Observational Studies

Randomized trials that directly compare empagliflozin and dapagliflozin in heart failure are lacking; therefore, comparative assessments rely on (1) network/meta-analytic pooling of randomized placebo-controlled trials, and (2) observational real-world cohort studies and registries that compare agents head-to-head. Both approaches have strengths and important limitations.

Meta-analyses and systematic reviews of randomized trials consistently support a class effect of SGLT2 inhibitors in reducing HF hospitalization and improving health-related quality of life across EF spectra. Recent comprehensive reviews that include EMPEROR-Preserved, DELIVER, DAPA-HF and EMPEROR-Reduced conclude that the magnitude of benefit on HF hospitalization is robust and similar across the major agents when trials are compared indirectly; some pooled analyses suggest small numerical differences in specific endpoints (for instance, trends favoring one agent for particular composite outcomes), but confidence intervals overlap and no high-quality randomized head-to-head evidence confirms superiority of one drug over another. Heterogeneity in trial populations, endpoint definitions (first vs. total events), and background therapy complicates indirect comparisons [28].

Observational and registry data provide direct comparisons but are subject to residual confounding despite adjustment techniques. Several recent large cohort analyses (including multi-center registries and claims-based studies) have compared cardiovascular and HF outcomes in patients started on empagliflozin versus dapagliflozin. Most report broadly comparable rates of HF hospitalization, cardiovascular events and all-cause mortality between the two agents after multivariable adjustment and propensity matching; a few studies have suggested small, heterogeneous differences in certain subgroups (for example, in patients with diabetes or chronic kidney disease CKD), but these findings have not been consistently replicated [29]. A notable recent JAMA Network cohort and large comparative-effectiveness analyses found no major differences in key long-term cardiovascular outcomes between the agents in real-world practice [30]. Nine randomized controlled trials comprising a total of 16,240 patients meeting the inclusion criteria were analyzed, with a mean age of 68.6 ± 9.9 years. Both dapagliflozin and empagliflozin were administered at a standardized dose of 10 mg. Empagliflozin demonstrated a superior improvement in overall Kansas City Cardiomyopathy Questionnaire (KCCQ) scores compared to dapagliflozin, with a standardized mean difference (SMD) of 1.59 (95% CI, 0.10 to 3.09; *p* < 0.03; I^2^ = 99.5%) at 6 months and 0.12 (95% CI, 0.08 to 0.16; *p* < 0.00001; I^2^ = 48%) at 1 year. Additionally, empagliflozin showed a trend toward improvement in the 6 min walk distance (6MWD); however, this difference did not reach statistical significance (SMD 0.49; 95% CI, −0.33 to 1.31; *p* = 0.24; I^2^ = 92.6%). Importantly, no significant differences were observed between empagliflozin and dapagliflozin in the incidence of major adverse events, including renal insufficiency and hypoglycemia [18].

When interpreting comparative studies caution required: confounding by indication (prescribers choose a specific agent for reasons linked to prognosis) can bias observational comparisons; differences in trial populations (e.g., EMPEROR vs. DELIVER vs DAPA-HF) and endpoint definitions make indirect randomized comparisons imperfect; safety signals reported in one drug’s development program (or in separate non-HF trials) may reflect population differences more than true drug differences; pharmacovigilance and pooled safety meta-analyses are therefore important complements to efficacy comparisons [27].

Pooled randomized evidence and most adjusted real-world comparative studies support the interpretation that empagliflozin and dapagliflozin have largely comparable efficacy for heart-failure outcomes at a population level. Small, context-specific differences reported in observational analyses merit further investigation but should not, at present, drive blanket preference for one agent over the other in typical HFmrEF/HFpEF/HFrEF patients—choice should instead consider renal function, tolerability, cost/access, and patient preferences. Future randomized head-to-head or pragmatic trials would be the ideal way to resolve remaining uncertainties.

## 4. Evidence in Acute Heart Failure

### Comparison of Empagliflozin and Dapagliflozin in Acute Heart Failure

The expanding role of SGLT2 inhibitors in the management of heart failure has prompted investigation into their potential benefits even during acute heart failure (AHF) hospitalizations. Two pivotal trials–EMPULSE and DICTATE-AHF–have specifically evaluated the early initiation of empagliflozin and dapagliflozin, respectively, in this high-risk population [31]. While both studies aim to assess safety and efficacy in the acute setting, they differ significantly in study design, patient population, endpoints, and interpretability of results [32,33].

The EMPULSE trial was a randomized, double-blind, placebo-controlled multicenter trial designed to provide robust evidence regarding the safety and efficacy of empagliflozin when initiated in patients hospitalized for AHF. By contrast, DICTATE-AHF was a randomized, open-label, exploratory pilot study, focusing primarily on surrogate endpoints related to volume management and diuretic response, rather than definitive clinical outcomes [32,33].

EMPULSE enrolled 530 patients hospitalized with acute heart failure, regardless of left ventricular ejection fraction (LVEF) or diabetes status. The trial population was broadly inclusive, reflecting real-world AHF presentations. In contrast, DICTATE-AHF was restricted to patients with type 2 diabetes mellitus, thereby narrowing its generalizability. Additionally, while EMPULSE included patients regardless of prior heart failure history, DICTATE-AHF enrolled patients with pre-existing heart failure and acute decompensation [32,33].

Both trials initiated SGLT2 inhibitor therapy during hospitalization, with EMPULSE starting empagliflozin at a median of 3 days post-admission, and DICTATE-AHF similarly targeting early in-hospital initiation of dapagliflozin. However, their clinical objectives diverged. EMPULSE aimed to assess overall clinical benefit, using a hierarchical composite endpoint encompassing mortality, heart failure events, and quality of life (via KCCQ). DICTATE-AHF focused on hemodynamic and decongestive parameters, such as diuretic efficiency, volume status, and natriuresis—making it more mechanistic in nature [32,33].

EMPULSE demonstrated a significant clinical benefit with empagliflozin over placebo, with a win ratio of 1.36 (*p* = 0.0054), and consistent effects across subgroups, including patients with preserved or reduced ejection fraction and those without diabetes. The trial provided strong support for the early in-hospital initiation of empagliflozin as both effective and safe in the AHF setting. Before EMPULSE trial 2 years ago the EMPA-RESPONSE-AHF trial investigated the use of empagliflozin in patients hospitalized with acute heart failure and demonstrated that the therapy was safe and well tolerated. Notably, empagliflozin was associated with a reduction in the composite outcome of worsening heart failure, heart failure rehospitalization, or all-cause mortality within 60 days of initiation [34].

In contrast, DICTATE-AHF, while not powered for clinical outcomes, showed favorable trends in decongestion metrics and volume status with dapagliflozin compared to standard care. However, the lack of blinding and modest sample size (~240 patients) limit the strength of its conclusions. Importantly, no safety concerns were observed, reinforcing the tolerability of early SGLT2 inhibitor initiation [33].

EMPULSE provides higher-grade evidence supporting the initiation of empagliflozin in a broad AHF population, demonstrating both symptomatic and clinical event reduction. In contrast, DICTATE-AHF offers exploratory insight into the physiologic effects of dapagliflozin during acute decompensation in diabetic patients. While promising, the findings from DICTATE-AHF warrant confirmation in larger, event-driven trials with rigorous clinical endpoints [32,33].

In summary, both EMPULSE and DICTATE-AHF contribute meaningfully to the evolving paradigm of SGLT2 inhibitor use in acute heart failure. EMPULSE stands out for its methodological rigor and clinical endpoint data, supporting empagliflozin as a viable in-hospital therapeutic strategy. DICTATE-AHF, although limited by its design and scope, lays the groundwork for future studies exploring dapagliflozin’s early hemodynamic benefits. Together, these trials suggest that timely initiation of SGLT2 inhibitors during acute heart failure hospitalization is both feasible and potentially beneficial, though further evidence is needed to define the role of dapagliflozin in this context.

DAPA-ACT HF-TIMI 68 is a phase 4, randomized, double-blind, placebo-controlled trial assessing the efficacy and safety of initiating dapagliflozin during hospitalization after clinical stabilization for AHF. The trial enrolled approximately 2400 patients and used a primary composite endpoint of cardiovascular death or worsening heart failure. While enrollment has been completed, results have not yet been reported at the time of writing. The study’s design allows evaluation of dapagliflozin in a setting that mirrors current guideline-directed therapy initiation patterns for other heart failure medications—specifically, after hemodynamic stability has been achieved [35].

When interpreted together DICTATE-AHF and DAPA-ACT HF-TIMI 68, these trials illustrate complementary aspects of dapagliflozin use in AHF: DICTATE-AHF evaluates very early initiation with a focus on short-term decongestion metrics, whereas DAPA-ACT HF-TIMI 68 assesses stabilized patients with a hard cardiovascular outcome composite. Pending the results of DAPA-ACT HF-TIMI 68, DICTATE-AHF suggests that early initiation may be safe and associated with more rapid decongestion, albeit without a statistically significant improvement in the primary efficiency measure.

## 5. Evidence Post Myocardial Infarction Heart Failure Prevention

Patients surviving an acute myocardial infarction (MI) remain at elevated risk for developing HF, recurrent ischemic events, and cardiovascular death despite optimal reperfusion and secondary prevention strategies. The post-MI period is characterized by adverse ventricular remodeling, neurohormonal activation, and heightened cardiorenal vulnerability—pathophysiological processes potentially modifiable by SGLT2 inhibition [36].

The EMPACT-MI and DAPA-MI trials are both clinical studies evaluating SGLT2 inhibitors in patients after acute myocardial infarction (AMI). While they share a common goal—investigating whether early initiation of SGLT2 inhibitors following MI improves clinical outcomes—they differ in design, endpoints, drug used, and study outcomes. These trials aim to extend the established benefits of SGLT2 inhibitors in chronic heart failure and diabetes to the high-risk post-infarction setting, a population traditionally vulnerable to adverse remodeling and subsequent heart failure development [37,38].

The DAPA-MI trial is a registry-based, pragmatic, randomized controlled trial conducted primarily in Sweden and the UK. It assessed whether dapagliflozin, initiated within 7 days of MI, could reduce biomarkers of heart failure and improve surrogate measures of cardiac function in a broad AMI population. Importantly, DAPA-MI did not use clinical outcomes such as mortality or hospitalization as primary endpoints, but instead focused on subclinical markers and modeling-based estimates of treatment benefit [38].

In contrast, EMPACT-MI was a conventional, event-driven, randomized controlled trial evaluating empagliflozin, also initiated early post-MI (within 14 days), but with a hard clinical primary outcome: a composite of all-cause mortality or hospitalization for heart failure. EMPACT-MI enrolled a higher-risk population with left ventricular dysfunction (LVEF ≤ 45%) or pulmonary congestion, thereby enriching for those most likely to benefit from heart failure prevention strategies [37].

DAPA-MI demonstrated modeled improvements in heart failure risk and biomarker profiles, suggesting a potential benefit of early dapagliflozin initiation, but did not measure or report actual clinical endpoints such as hospitalization or mortality. It supported feasibility and safety but remained exploratory [38].

EMPACT-MI, in contrast, did not show a statistically significant reduction in its composite endpoint of all-cause death or hospitalization for heart failure. However, numerical trends favored empagliflozin, and the trial confirmed the safety and tolerability of SGLT2 inhibition shortly after MI in patients with LV dysfunction. The relatively low event rates observed in EMPACT-MI may be partly attributable to the high quality of post-AMI care, including prompt revascularization and comprehensive secondary prevention strategies. Notably, 90% of participants underwent revascularization, 98% received antiplatelet therapy, and 90% were treated with statins, reflecting adherence to guideline-directed medical therapy. However, several limitations warrant consideration. First, clinical endpoints were not centrally adjudicated, which may introduce variability in outcome reporting. Second, the trial did not include outpatient heart failure events as part of the clinical endpoint framework, potentially underestimating the total burden of heart failure exacerbations. Third, there was limited representation of women, older adults, and ethnic minority populations, which may affect the generalizability of the findings [37].

The main difference lies in the type of data provided: DAPA-MI supports a hypothesis-generating, model-based benefit from dapagliflozin in a broad MI cohort, while EMPACT-MI delivers hard clinical outcome data but in a higher-risk population, and the results were neutral for the primary endpoint. Together, these trials underscore both the promise and the limitations of SGLT2 inhibitors in the acute coronary syndrome setting.

The implications for secondary prevention are twofold. First, the absence of definitive benefit for major cardiovascular endpoints in either trial suggests that SGLT2 inhibitors are not yet established as routine therapy for all post-MI patients without HF. Second, both trials indicate that early initiation is safe and may confer favorable effects on surrogate markers, HF risk, and cardiometabolic health, supporting further investigation in high-risk subgroups. In patients with reduced LVEF, clinical congestion, or other predictors of HF after MI, SGLT2 inhibitors may have a role in attenuating remodeling and preventing progression to symptomatic HF, aligning with their proven efficacy in established HF populations. Ongoing analyses and longer-term follow-up will be essential to determine whether early post-MI SGLT2 inhibition should be integrated into standard secondary prevention regimens beyond current guideline-directed therapies [37,38,39].

Table 2 provides a comprehensive overview of the key randomized clinical trials evaluating SGLT2 inhibitors in various heart failure populations, summarizing essential trial characteristics, inclusion criteria, primary endpoints, and major outcome findings.

## 6. Safety Considerations

### 6.1. Impact on Renal Function for Patient with HF Treated with SGLT2-i

SGLT2 inhibitors reduce sodium and glucose reabsorption in the proximal tubules, leading to glucosuria, natriuresis, and reduced intraglomerular pressure via tubuloglomerular feedback. Although initially raising concerns about volume depletion and acute kidney injury, clinical trials have shown that these agents actually lower the risk of acute kidney injury and provide long-term renal protection by slowing the decline in kidney function [41].

A post hoc analysis of over 11,000 participants from the DAPA-HF and DELIVER trials examined the incidence of eGFR decline to <25 mL/min/1.73 m^2^. Only 3.2% of patients experienced such deterioration, mostly occurring after the first month. The risk of eGFR decline was similar between dapagliflozin and placebo groups. Importantly, dapagliflozin reduced the risk of the primary composite outcome regardless of whether patients experienced kidney function deterioration, suggesting renal decline did not diminish the overall cardiovascular benefit of the drug [42].

Although 30–40% of patients on SGLT2 inhibitors experience an initial eGFR decline ≥10 mL/min/1.73 m^2^, and a smaller proportion (3–6%) progress to CKD stage G4, such declines are generally well tolerated. Evidence from DAPA-HF and DELIVER suggests that these changes are often part of the natural disease course rather than a drug-induced effect. Importantly, continuing SGLT2 inhibitors despite eGFR decline is associated with better long-term outcomes, especially in sicker patients who may benefit the most [2,5].

Despite familiarity with SGLT2 inhibitors’ effects on intraglomerular pressure, acute or chronic eGFR declines—particularly below 25 mL/min/1.73 m^2^—may prompt unnecessary discontinuation in clinical practice due to “hypercreatinemia-phobia” [43].

However, in trials like DAPA-HF and DELIVER, treatment was generally continued despite such declines, with 75% of patients remaining on therapy. Safety outcomes remained consistent across eGFR strata, and no subgroup has yet shown a risk-benefit profile that argues against continuing SGLT2 inhibitors. These findings support a permissive approach to eGFR decline in high-risk patients, aligning with KDIGO guidelines that endorse continuation even below 20 mL/min/1.73 m^2^ [2,5].

Regarding renal outcomes, both DAPA-HF and EMPEROR-Reduced demonstrated that SGLT2 inhibitors significantly slowed the decline in eGFR over time. However, while the composite kidney outcome reached statistical significance in EMPEROR-Reduced, it did not in DAPA-HF. This discrepancy may be attributed to the lower number of renal events in DAPA-HF, likely influenced by higher baseline eGFR thresholds for trial inclusion and the use of a more stringent definition for kidney events—requiring a sustained 50% decline in eGFR, compared to 40% in EMPEROR-Reduced [2,3,23].

### 6.2. Diuretic Effect of SGLT2i in Patient with HF

A comparative analysis of dapagliflozin and empagliflozin in the context of acute heart failure (AHF) reveals notable distinctions in their diuretic profiles and clinical impact during early decompensation management.

Dapagliflozin, administered at a dose of 10 mg, demonstrated evidence of enhanced diuresis in patients with AHF, despite not achieving a statistically significant improvement in weight-based diuretic efficiency [44]. This suggests that while dapagliflozin supports natriuresis, its incremental benefit in optimizing diuretic response may be modest under standard dosing conditions [39]. Conversely, the EMPAG-HF study investigated empagliflozin at a higher dose of 25 mg, initiated within 12 h of hospital admission for acute decompensated heart failure. Empagliflozin was associated with a 25% increase in urine output when added to conventional loop diuretics, without adversely affecting the eGFR slope. Additionally, empagliflozin improved diuretic efficiency, promoted greater reductions in NT-proBNP levels, and showed a trend toward body weight reduction. The choice of a higher dose in this setting was intentional, aiming to amplify its diuretic effects and assess tolerability in combination with high-intensity heart failure therapies [45].

Collectively, these findings suggest that while both agents appear safe and beneficial in the acute heart failure setting, empagliflozin—particularly at higher doses—may offer superior early decongestive efficacy and biomarker reduction. Nonetheless, differences in study design, dosing strategies, and patient populations warrant cautious interpretation [46].

### 6.3. Impact of Treatment with SGLT2-i on Hyperkaliemia in Patient with HF

The comparative risk of hyperkalemia between empagliflozin and dapagliflozin in patients with HF, regardless of ejection fraction phenotype, has been a subject of growing clinical interest, particularly given the widespread use of renin–angiotensin–aldosterone system inhibitors (RAASi), which increase the risk of hyperkalemia. Both empagliflozin and dapagliflozin appear to exert beneficial effects on potassium homeostasis; however, subtle distinctions may exist between the two agents.

Across major clinical trials—including EMPEROR-Reduced and EMPEROR-Preserved for empagliflozin, and DAPA-HF and DELIVER for dapagliflozin—both drugs were associated with either a neutral or protective effect against hyperkalemia. In these studies, patients receiving SGLT2 inhibitors had lower rates of moderate-to-severe hyperkalemia compared to placebo, despite the concomitant use of RAASi and mineralocorticoid receptor antagonists (MRAs) [2,3,5,24]. This potassium-sparing effect is thought to be mediated through enhanced distal sodium delivery and mild volume contraction, leading to improved aldosterone activity and urinary potassium excretion.

Specifically, the EMPEROR trials noted a consistent reduction in investigator-reported hyperkalemia events with empagliflozin. In EMPEROR-Reduced, empagliflozin was associated with a lower incidence of hyperkalemia compared to placebo, particularly among those on MRAs [47]. Similarly, DAPA-HF and DELIVER showed a reduced or stable risk of hyperkalemia with dapagliflozin use, and in some subgroup analyses, dapagliflozin appeared to reduce the need for MRA dose down-titration due to potassium elevation. Importantly, no head-to-head comparisons have been conducted to directly contrast the effects of empagliflozin versus dapagliflozin on serum potassium levels in heart failure populations [2,5]. Nevertheless, the available evidence suggests that both agents may confer a class effect in mitigating the risk of hyperkalemia, with comparable safety profiles. Given the clinical relevance of hyperkalemia as a limiting factor for optimal guideline-directed medical therapy, especially in patients with chronic kidney disease or advanced HF, the use of either empagliflozin or dapagliflozin may support better treatment adherence and outcomes through improved electrolyte balance.

Greene et al. [48] recently proposed that combining SGLT2 inhibitors with angiotensin receptor–neprilysin inhibitors (ARNIs) may lower the risk of hyperkalemia and reduce the likelihood of discontinuing mineralocorticoid receptor antagonists (MRAs). This combination may enhance the ability to up-titrate guideline-directed medical therapy (GDMT). The authors argue that hyperkalemia should no longer be a major deterrent to optimizing therapy, as it can be effectively prevented and managed.

In conclusion, while both empagliflozin and dapagliflozin demonstrate favorable potassium safety profiles in patients with heart failure, existing data do not indicate a clinically meaningful difference between the two in terms of hyperkalemia risk.

### 6.4. Genital Infections

In the context of HF management, both empagliflozin and dapagliflozin are associated with an increased risk of genital infections, a class effect of these agents due to glucosuria. However, comparative data suggest subtle differences in incidence rates that may be clinically relevant, especially in susceptible populations. Empagliflozin has been associated with a slightly higher rate of genital mycotic infections in clinical trials, particularly in female patients, although these infections were generally mild to moderate in severity and responded well to standard antifungal treatment. For instance, in the EMPEROR-Reduced and EMPEROR-Preserved trials, genital infections were reported more frequently in the empagliflozin arm than placebo, but did not lead to significant discontinuation rates. Dapagliflozin, likewise, has shown a modest increase in genital infections in trials such as DAPA-HF and DELIVER [2,3,5,24]. However, some analyses indicate a slightly lower incidence of genital infections compared to empagliflozin. This difference may relate to pharmacokinetic and pharmacodynamic variations, such as differences in urinary glucose excretion or tissue distribution, though direct head-to-head comparisons in HF populations are lacking.

The reported incidence of severe or complicated urinary tract infections (UTIs) across clinical trials of SGLT2 inhibitors varies, in part due to differing definitions applied within studies. Typically, “severe UTI” in these trials refers to UTIs or pyelonephritis that directly result in hospitalization, as well as UTIs complicated by sepsis (i.e., urosepsis). In large cardiovascular outcome trials involving SGLT2 inhibitors, the incidence of such events has consistently remained low, with rates under 2% across all studies, with no difference between two drugs [49].

A meta-analysis of smaller randomized controlled trials further supports these findings, demonstrating a pooled incidence of 0.3% for both pyelonephritis and urosepsis. Importantly, no statistically significant difference was observed between SGLT2 inhibitors and control groups for either pyelonephritis (risk ratio [RR]: 0.78; 95% confidence interval [CI]: 0.52–1.18) or urosepsis (RR: 1.03; 95% CI: 0.96–1.11). These data suggest that while vigilance for severe UTI is warranted, SGLT2 inhibitors do not meaningfully increase the risk of these complications in clinical practice [50].

Importantly, the risk of genital infections with either agent does not appear to be significantly modified by HF phenotype (i.e., HFrEF vs. HFpEF) and remains consistent with what is observed in type 2 diabetes populations. Preventive measures, including patient education on hygiene and early symptom recognition, remain key to minimizing this adverse effect and maintaining adherence to therapy.

In summary, while both empagliflozin and dapagliflozin carry a small but identifiable risk for genital infections in HF patients, there is no conclusive evidence to favor one over the other solely on this basis. The benefits of SGLT2 inhibitors in reducing HF morbidity and mortality outweigh the manageable risk of these infections in most patients.

Fournier’s gangrene is a rare but life-threatening form of necrotizing fasciitis involving the superficial and deep tissues of the perineal and genital regions [51]. Despite initial safety concerns, the current evidence does not establish a causal relationship between sodium-glucose cotransporter-2 inhibitors (SGLT2is) and this condition [52]. A meta-analysis of randomized controlled trials including 69,573 participants reported only three cases of Fournier’s gangrene in the SGLT2i group and six cases in the control group. Similarly, data from a large U.S. Veterans Health Administration cohort found an incidence rate of 1.14 cases per 1000 patient-years among individuals receiving SGLT2is, with no significant difference in risk when compared to patients treated with GLP-1 receptor agonists. Although a rise in case reports has been observed following the FDA’s safety alert, these do not confirm a direct causal link. Thus, while vigilance remains important, the absolute risk of Fournier’s gangrene associated with SGLT2i therapy appears to be exceedingly low [53].

### 6.5. Euglycemic Diabetic Ketoacidosis in Heart Failure Patients

Euglycemic diabetic ketoacidosis (euDKA) is an uncommon but recognized adverse effect of SGLT2 inhibitors, characterized by elevated serum ketones and metabolic acidosis in the presence of only mild or absent hyperglycemia. Although the condition is more frequently reported in patients with type 1 diabetes, it can also occur in those with type 2 diabetes and, rarely, in non-diabetic individuals under specific stressors such as acute illness, prolonged fasting, or perioperative states [54]. In the context of heart failure (HF) management, both empagliflozin and dapagliflozin have been studied extensively across HFrEF, HFmrEF, and HFpEF populations, with trial safety data providing insight into the incidence of euDKA in these settings [55,56].

Across major randomized HF trials—DAPA-HF, DELIVER, EMPEROR-Reduced, and EMPEROR-Preserved—the absolute incidence of euDKA was extremely low (<0.1–0.3% over median follow-up periods of 1.5–2.3 years) and did not differ meaningfully between empagliflozin and dapagliflozin [2,3,5,24]. Most events occurred in participants with pre-existing diabetes, often in the context of precipitating factors such as reduced oral intake, infection, or aggressive diuresis. In pooled analyses, there was no statistically significant excess risk compared to placebo in non-diabetic HF participants, underscoring that baseline glycemic status is a major determinant of risk. Observational data from real-world HF cohorts similarly suggest that the risk of euDKA in this population is rare and clinically manageable with appropriate patient selection and education.

Importantly, no head-to-head HF trial has compared empagliflozin and dapagliflozin with euDKA as a primary or prespecified safety endpoint, and comparative pharmacovigilance studies in HF have not demonstrated a consistent signal favoring one agent over the other. Both drugs share a similar mechanism of glycosuria-induced reduction in circulating insulin and increased glucagon, which can promote ketogenesis under stress [53]. Therefore, current expert consensus emphasizes that mitigation strategies—such as pausing therapy during acute illness or perioperative periods, maintaining adequate caloric and carbohydrate intake, and educating patients about early symptom recognition—are equally applicable regardless of which SGLT2 inhibitor is prescribed in HF.

From a practical standpoint, the exceedingly low event rates in large-scale HF trials and the absence of major differences between empagliflozin and dapagliflozin support the use of either agent in appropriate HF patients, while remaining vigilant for euDKA in those with diabetes or other predisposing conditions.

To summarize, SGLT2 inhibitors have demonstrated a generally favorable safety profile in patients with heart failure across phenotypes. Adverse events commonly attributed to this drug class include genital mycotic infections, transient declines in eGFR, volume depletion, and, more rarely, euglycemic diabetic ketoacidosis (euDKA). Notably, the absolute risk of genital infections ranges between 4 and 6% in major trials (e.g., DAPA-HF, EMPEROR-Reduced), typically presenting as mild and responsive to treatment, and more often observed in female patients. While slight variations in incidence have been noted between empagliflozin and dapagliflozin, current data do not support a clinically meaningful difference, and these are best interpreted as class effects [57].

In terms of renal safety, acute eGFR reductions of ≥10 mL/min/1.73m^2^ occur in 30–40% of patients but are transient in most cases. Only ~3% of patients experienced a decline to <25 mL/min/1.73m^2^ in pooled post hoc analyses of DAPA-HF and DELIVER, with no difference between treatment and placebo arms. Importantly, renal decline did not attenuate cardiovascular benefits, and trial protocols generally continued therapy despite moderate renal impairment, reinforcing the need to avoid unnecessary discontinuation [57].

The risk of hyperkalemia was lower or neutral in SGLT2 inhibitor arms versus placebo, even in patients receiving RAAS inhibitors or MRAs. While no head-to-head comparison exists, this potassium-sparing effect is considered a class characteristic, likely mediated through enhanced distal sodium delivery and improved aldosterone signaling [58].

The incidence of euDKA in heart failure trials was exceedingly low, typically <0.3%, and occurred almost exclusively in patients with diabetes and precipitating factors (e.g., infection, fasting). No significant difference in euDKA rates has been observed between empagliflozin and dapagliflozin, suggesting a shared mechanism involving glycosuria-induced shifts in insulin/glucagon balance [59].

Finally, severe urinary tract infections and Fournier’s gangrene are rare, with pooled meta-analyses showing no increased incidence versus placebo. Although early safety warnings were issued for Fournier’s gangrene, current evidence does not establish causality [60].

Overall, the adverse effects of SGLT2 inhibitors in heart failure are mostly predictable, infrequent, and manageable. The data to date support a class-wide safety profile, with no robust evidence favoring one agent over another for any specific adverse outcome. Where observed differences exist, they remain hypothesis-generating and should be interpreted with caution until confirmed by direct comparative trials.

## 7. Cost-Effectiveness and Health Policy Considerations

### 7.1. Cost per QALY Gained

Cost-effectiveness analyses (CEA) of SGLT2 inhibitors in heart failure consistently report favorable incremental cost-effectiveness ratios (ICERs) relative to commonly accepted willingness-to-pay thresholds in high-income countries. In modeling studies using data from DAPA-HF, DELIVER, EMPEROR-Reduced, and EMPEROR-Preserved, both dapagliflozin and empagliflozin produce incremental gains of approximately 0.10–0.30 quality-adjusted life years (QALYs) over a patient’s lifetime horizon, driven largely by reduced hospitalizations for heart failure and associated mortality benefits [61,62,63]. Notably, indirect comparisons suggest broadly similar cost per QALY for both empagliflozin and dapagliflozin, with differences primarily driven by negotiated pricing and local reimbursement structures rather than intrinsic differences in efficacy or safety.

### 7.2. Impact on Hospitalization Costs

The most immediate economic impact of SGLT2 inhibitor therapy in HF is the reduction in all-cause and HF-specific hospitalizations, which account for a substantial proportion of the direct costs of HF care. Data from large randomized controlled trials and real-world registries demonstrate a relative reduction in first and total HF hospitalizations of 25–30% with either empagliflozin or dapagliflozin, translating into meaningful cost savings for health systems. While absolute savings depend on hospitalization unit costs and baseline event rates, the net budget impact is generally favorable when therapy is targeted to high-risk populations, such as those with recent hospitalization or advanced NYHA functional class. Importantly, the cost benefits accrue early—within the first year of therapy—reinforcing the rationale for prompt initiation after HF diagnosis or decompensation [64,65,66].

### 7.3. Access, Reimbursement, and Guideline Recommendations

Despite robust evidence for clinical benefit and favorable cost-effectiveness profiles, access to SGLT2 inhibitors for HF remains variable worldwide. In many high-income countries, both empagliflozin and dapagliflozin are included in reimbursement lists for HFrEF, and guideline recommendations (ESC, AHA/ACC/HFSA) position them as foundational therapy irrespective of diabetes status. However, formulary restrictions, prior authorization requirements, and copay burdens still limit uptake, particularly in lower-income and middle-income settings. Differential pricing agreements and tiered reimbursement policies often influence agent selection more than clinical considerations. For HFpEF and HFmrEF, the most recent ESC and American College of Cardiology (ACC) guidelines endorse either empagliflozin or dapagliflozin based on EMPEROR-Preserved and DELIVER, though some payers have yet to update coverage to reflect these indications. In the context of universal health coverage systems, centralized negotiation can substantially lower acquisition costs, improving equity of access. Policy strategies to maximize uptake include inclusion in essential medicines lists, expansion of coverage for non-diabetic HF indications, and integration of SGLT2 inhibitors into bundled payment or value-based care models that reward hospitalization reduction [66,67].

Economic evaluations consistently find that adding either empagliflozin or dapagliflozin to standard heart-failure care is cost-effective in many high-income health systems, primarily through reductions in HF hospitalizations that translate into early and ongoing cost offsets. Differences between the two agents in modeled economic outcomes are usually marginal and driven more by local acquisition price and reimbursement policies than by efficacy or safety differences observed in clinical trials; consequently, policy efforts that lower acquisition cost or expand coverage will have greater impact on population uptake than head-to-head clinical superiority [30].

## 8. Future Directions and Limitations of the Study

The clinical development of empagliflozin and dapagliflozin in HF reflects both overlapping and distinct strategic priorities shaped by differences in timing, population targeting, and combination approaches. Both agents have established efficacy across the HF spectrum—chronic HFrEF, HFmrEF, and HFpEF—yet ongoing and upcoming trials emphasize complementary gaps.

Dapagliflozin’s development program has been notable for early focus on acute heart failure and very early initiation, as evidenced by trials such as DAPA-ACT HF-TIMI-68 and DICTATE-AHF, which examine initiation shortly after hospitalization or during acute decompensation. DAPA-ACT HF-TIMI 68, a large-scale, event-driven trial, is evaluating the clinical benefit of initiating dapagliflozin after hemodynamic stabilization in patients hospitalized for acute heart failure. The trial’s design, which mirrors real-world clinical decision-making, is likely to provide pivotal evidence supporting early post-acute implementation of SGLT2i therapy [35]. This suggests a strategic prioritization on expanding SGLT2i use into the acute HF setting, an area where evidence has historically been limited. Furthermore, dapagliflozin’s combination trials, such as SOGALDI-PEF (dapagliflozin plus spironolactone) and other pragmatic co-prescription registries with GLP-1 receptor agonists signal a forward-looking approach aimed at targeting multifactorial pathophysiology by layering SGLT2 inhibition on established neurohormonal blockade or metabolic therapy [68,69].

In contrast, empagliflozin’s HF development program has prominently included the EMPACT-MI trial, which investigates very early initiation post-myocardial infarction to prevent incident HF and reduce adverse remodeling. This focus on primary prevention of HF post-MI differentiates empagliflozin’s strategy by aiming upstream at interrupting disease progression at its earliest clinical junction. While combination trials for empagliflozin are less extensively publicized, ongoing studies such as the finerenone plus empagliflozin trial suggest a growing interest in synergistic neurohormonal blockade and cardiorenal protection [70]. Given empagliflozin’s robust renal and cardiovascular safety data, further combination trials—potentially mirroring the design philosophy of BaxDuo Prevent-HF, (a fixed-dose combination of dapagliflozin and a nonsteroidal mineralocorticoid receptor antagonist)—may be anticipated, capitalizing on integrated pharmacologic approaches to maximize prevention and treatment of HF [71].

Emerging evidence supports the integration of SGLT2 inhibitors into multidrug regimens for heart failure, particularly in combination with other disease-modifying agents such as angiotensin receptor–neprilysin inhibitors (ARNIs) and mineralocorticoid receptor antagonists (MRAs). These combinations offer complementary pathophysiological coverage: while ARNIs enhance natriuretic peptide signaling and MRAs attenuate aldosterone-mediated fibrosis and inflammation, SGLT2 inhibitors uniquely modulate interstitial volume, sympathetic activity, and myocardial metabolism. Recent registry analyses and post hoc trial data suggest that co-administration of SGLT2 inhibitors with ARNIs or MRAs may not only improve tolerability (e.g., through mitigation of hyperkalemia risk) but also enable more effective up-titration of guideline-directed medical therapy (GDMT) by stabilizing renal function and volume status [72].

The BaxDuo Prevent-HF trial epitomizes the emerging paradigm of combining SGLT2 inhibitors with novel agents such as nonsteroidal mineralocorticoid receptor antagonists (nsMRAs) to target complementary pathways. It represents a clear evolution beyond monotherapy, aiming to leverage additive or synergistic benefits in HF prevention and progression delay. Although no identical empagliflozin-fixed combination trials have been published to date, ongoing empirical studies of empagliflozin in combination with nsMRAs or spironolactone suggest that such a strategy is imminent in the empagliflozin portfolio, reflecting convergent development priorities [71].

**Figure 1:**
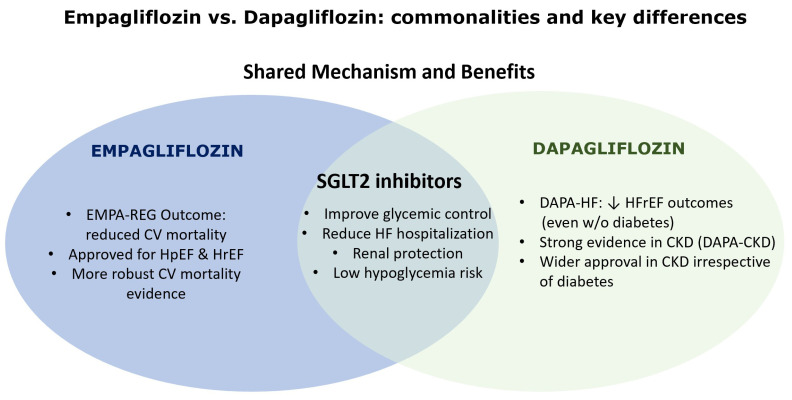
Commonalities and key differences empagliflozin versus dapagliflozin—current state of knowledge. ↓ means reduction.

In summary, dapagliflozin is leading with pragmatic and acute HF initiation trials alongside early combination therapy exploration, whereas empagliflozin focuses strongly on early post-MI prevention with growing emphasis on combination approaches targeting cardiorenal axes. Both agents are likely to expand indications through trials that refine patient selection, timing, and multi-drug regimens, ultimately moving toward personalized HF therapy informed by underlying pathophysiology and comorbidity burden. Figure 1 is presenting the current state of knowledge regarding the commonalities and key differences between empagliflozin versus dapagliflozin.

Several limitations should be acknowledged when interpreting the findings presented in this review. Firstly, although apparent differences between dapagliflozin and empagliflozin in specific heart failure phenotypes (HFrEF vs. HFpEF) are discussed, these should be interpreted with caution. Such distinctions likely reflect differences in trial design, patient selection, baseline characteristics, and endpoints, rather than definitive pharmacodynamic superiority. In the absence of direct comparative (head-to-head) trials, any suggestions of agent-specific advantages remain speculative and should be regarded as hypothesis-generating.

Secondly, while emerging data suggest potential mechanistic distinctions—such as greater anti-fibrotic signaling with empagliflozin or enhanced vascular and endothelial modulation with dapagliflozin—these remain predominantly supported by preclinical or early-phase translational studies. As such, these proposed mechanisms should be interpreted as preliminary and not yet confirmed in large-scale clinical investigations. Accordingly, we have taken care to distinguish established outcome data from experimental or emerging mechanistic hypotheses throughout the manuscript.

## 9. Conclusions

This comprehensive review underscores the transformative impact of SGLT2 inhibitors, specifically empagliflozin and dapagliflozin, in the management of heart failure across its diverse phenotypes, including chronic, acute, post-myocardial infarction, and preserved or mildly reduced ejection fraction subgroups. Both agents consistently reduce composite end-point-heart failure hospitalization and cardiovascular mortality, reaffirming their status as foundational therapies.

However, key differences in their clinical development and emerging evidence highlight nuanced distinctions with important clinical implications. Both-empagliflozin and dapagliflozin represent complementary pillars in modern heart failure care, each with unique pharmacologic and clinical advantages. Tailoring agent selection to patient-specific factors, clinical setting, and emerging evidence will maximize the benefits of SGLT2 inhibition in improving heart failure outcomes.

## Figures and Tables

**Table 1:** Summary of key differences in mechanisms of action of empagliflozin and dapagliflozin.

Feature	Empagliflozin	Dapagliflozin
SGLT2 Selectivity	Highly selective for SGLT2, stronger potency in inhibiting glucose reabsorption [13,14]	Highly selective for SGLT2, slightly lower potency [15]
Potency	Greater potency leading to stronger glucose and sodium excretion [13,14]	Slightly lower potency, but still effective for heart failure [15]
Half-life	~12 h, once-daily dosing [16]	~10–12 h, once-daily dosing [17]
Kidney Function Effects	Stronger diuretic effects, more significant blood pressure reduction [4,16]	Similar diuretic effects, but slightly less pronounced blood pressure reduction [9,17]
Cardiovascular Effects	May have stronger myocardial and fibrosis effects, contributing to improved symptoms in HFrEF [18,20]	Stronger impact on vascular stiffness and diastolic dysfunction in HFpEF [5,21]
Heart Failure Impact	Potentially greater improvement in symptoms and NYHA class in HFrEF [3,8,20]	Greater efficacy in HFpEF in reducing hospitalizations and mortality [2,5]

**Table 2:** Key Randomized Controlled Trials of SGLT2 Inhibitors in Heart Failure: Design, Population, and Clinical Outcomes.

Trial Name & Drug	Sample Size	EF Inclusion Criteria	Median Follow-Up	Primary Endpoint	Key Baseline Characteristics	Main Outcome Results
DAPA-HF [2] (dapagliflozin)	4744	LVEF ≤ 40%	18.2 months	CV death or worsening HF, all-cause mortality	Mean LVEF: 31%, Median NT-proBNP: ~1430 pg/mL, 47% with prior HF hospitalization	↓26% primary endpoint, ↓CV and all-cause mortality
EMPEROR-Reduced [3] (empagliflozin)	3730	LVEF ≤ 40%	16 months	CV death or HF hospitalization	Mean LVEF: 27%, Median NT-proBNP: ~1900 pg/mL, 31% prior HF hospitalization	↓25% primary endpoint, nonsignificant ↓CV mortality
DELIVER [5] (dapagliflozin)	6263	LVEF > 40%	2.3 years	CV death or HF hospitalization	HFpEF + HFimpEF, broader population, higher baseline event risk	↓18% primary endpoint, no sig. CV death, exploratory ↓all-cause mortality
EMPEROR-Preserved [40] (empagliflozin)	5988	LVEF > 40%	26.2 months	CV death or HF hospitalization	Excluded recent HF hospitalization, lower risk cohort	↓21% primary endpoint, driven by HHF reduction, no ↓CV mortality
EMPULSE [32] (empagliflozin)	530	Any EF	90 days	Clinical benefit composite (death, HF events, QoL)	Included de novo and decompensated chronic HF, with or without diabetes	Win ratio 1.36 (*p* = 0.005), benefit across EF and diabetes status
DICTATE-AHF [33] (dapagliflozin)	240	Any EF	In-hospital	Decongestion metrics (diuretic response)	Patients hospitalized with acute decompensated chronic HF, 71% with T2DM	Improved natriuresis, exploratory study, no mortality data
EMPACT-MI [37] (empagliflozin)	6522	LVEF ≤ 45% or pulmonary congestion	1.5 years	Composite of first HF hospitalization or all-cause mortality	Post-MI patients, revascularized, LV dysfunction	Neutral primary composite, favorable trend driven by fewer HF hospitalizations and safe to start early after MI
DAPA-MI [38] (dapagliflozin)	4017	Any EF	11 months (median registry follow-up)	Biomarker-based surrogate endpoints	Post-MI, revascularized, early initiation within 7 days, without diabetes or prior HF	Modeled benefit only, no hard clinical outcomes

↓ means reduction.

## Data Availability

No new data were created or analyzed in this study. Data sharing is not applicable to this article.

## Abbreviations

The following abbreviations are used in this manuscript:

HF	Heart Failure
HFrEF	Heart Failure with Reduced Ejection Fraction
HFpEF	Heart Failure with Preserved Ejection Fraction
HFmrEF	Heart Failure with Mildly Reduced Ejection Fraction
SGLT2	Sodium-Glucose Cotransporter-2
T2DM	Type 2 Diabetes Mellitus
NT-proBNP	N-terminal pro-B-type Natriuretic Peptide
eGFR	Estimated Glomerular Filtration Rate
KCCQ	Kansas City Cardiomyopathy Questionnaire
6MWD	6-Minute Walk Distance
RAASi	Renin–Angiotensin–Aldosterone System Inhibitor
MRA	Mineralocorticoid Receptor Antagonist
ARNI	Angiotensin Receptor–Neprilysin Inhibitor
AHF	Acute Heart Failure
MI	Myocardial Infarction
LV	Left Ventricle
LVEF	Left Ventricular Ejection Fraction
QALY	Quality-Adjusted Life Year
ICER	Incremental Cost-Effectiveness Ratio
NYHA	New York Heart Association
GDMT	Guideline-Directed Medical Therapy
euDKA	Euglycemic Diabetic Ketoacidosis
UTI	Urinary Tract Infection
nsMRA	Nonsteroidal Mineralocorticoid Receptor Antagonist

## References

[1] Ziaeian B., Fonarow G.C. (2016). Epidemiology and aetiology of heart failure. Nat. Rev. Cardiol..

[2] McMurray J.J.V., Solomon S.D., Inzucchi S.E., Køber L., Kosiborod M.N., Martinez F.A., Ponikowski P., Sabatine M.S., Anand I.S., Bělohlávek J. (2019). DAPA-HF Trial Committees and Investigators. Dapagliflozin in patients with heart failure and reduced ejection fraction. N. Engl. J. Med..

[3] Butler J., Anker S.D., Filippatos G., Khan M.S., Ferreira J.P., Pocock S.J., Giannetti N., Januzzi J.L., Pina I.L., Lam C.S.P. (2021). Empagliflozin and health-related quality of life outcomes in patients with heart failure with reduced ejection fraction: The EMPEROR-Reduced trial. Eur. Heart J..

[4] Zannad F., Ferreira J.P., Pocock S.J., Zeller C., Anker S.D., Butler J., Filippatos G., Hauske S.J., Brueckmann M., Pfarr E. (2021). Cardiac and kidney benefits of empagliflozin in heart failure across the spectrum of kidney function: Insights from EMPEROR-Reduced. Circulation.

[5] Solomon S.D., de Boer R.A., DeMets D., Hernandez A.F., Inzucchi S.E., Kosiborod M.N., Lam C.S.P., Martinez F., Shah S.J., Lindholm D. (2021). Dapagliflozin in heart failure with preserved and mildly reduced ejection fraction: Rationale and design of the DELIVER trial. Eur. J. Heart Fail..

[6] Gallo L.A., Wright E.M., Vallon V. (2015). Probing SGLT2 as a therapeutic target for diabetes: Basic physiology and consequences. Diabetes Vasc. Dis. Res..

[7] Ferrannini E., Berk A., Hantel S., Pinnetti S., Hach T., Woerle H.J., Broedl U.C. (2013). Long-term safety and efficacy of empagliflozin, sitagliptin, and metformin: An active-controlled, parallel-group, randomized, 78-week open-label extension study in patients with type 2 diabetes. Diabetes Care.

[8] Zinman B., Wanner C., Lachin J.M., Fitchett D., Bluhmki E., Hantel S., Mattheus M., Devins T., Johansen O.E., Woerle H.J. (2015). Empagliflozin, Cardiovascular Outcomes, and Mortality in Type 2 Diabetes. N. Engl. J. Med..

[9] Vallon V., Thomson S.C. (2017). Targeting renal glucose reabsorption to treat hyperglycaemia: The pleiotropic effects of SGLT2 inhibition. Diabetologia.

[10] Trum M., Riechel J., Wagner S. (2021). Cardioprotection by SGLT2 Inhibitors-Does It All Come Down to Na+?. Int. J. Mol. Sci..

[11] Preda A., Montecucco F., Carbone F., Camici G.G., Lüscher T.F., Kraler S., Liberale L. (2024). SGLT2 inhibitors: From glucose-lowering to cardiovascular benefits. Cardiovasc. Res..

[12] Matteucci A., Pandozi C., Bonanni M., Mariani M.V., Sgarra L., Nesti L., Pierucci N., La Fazia V.M., Lavalle C., Nardi F. (2025). Impact of Empagliflozin and Dapagliflozin on Sudden Cardiac Death: A Systematic Review and Meta-Analysis of Adjudicated Randomized Evidence. Heart Rhythm..

[13] Heise T., Seewaldt-Becker E., Macha S., Hantel S., Pinnetti S., Seman L., Woerle H.J. (2013). Safety, tolerability, pharmacokinetics and pharmacodynamics following 4 weeks’ treatment with empagliflozin once daily in patients with type 2 diabetes. Diabetes Obes. Metab..

[14] Volino L., Pan E., Mansukhani R. (2014). Sodium Glucose Co-Transporter 2 (SGLT2) Inhibitors in Type 2 Diabetes: A Literature Review of Approved Products. Pharmacol. Pharm..

[15] Santer R., Calado J. (2010). Familial renal glucosuria and SGLT2: From a mendelian trait to a therapeutic target. Clin. J. Am. Soc. Nephrol..

[16] Macha S., Rose P., Mattheus M., Cinca R., Pinnetti S., Broedl U.C., Woerle H.J. (2014). Pharmacokinetics, safety and tolerability of empagliflozin, a sodium glucose cotransporter 2 inhibitor, in patients with hepatic impairment. Diabetes Obes. Metab..

[17] Kasichayanula S., Liu X., Lacreta F., Griffen S.C., Boulton D.W. (2014). Clinical pharmacokinetics and pharmacodynamics of dapagliflozin, a selective inhibitor of sodium-glucose co-transporter type 2. Clin. Pharmacokinet..

[18] Addo B., Ibrahim S., Akpan I., Ansari F., Allihien S.M. (2025). Comparative Efficacy Between Dapagliflozin and Empagliflozin on Symptoms and Functional Capacity in Patients with Heart Failure: A Network Meta-analysis. J. Card. Fail..

[19] Verma S., Rawat S., Ho K.L., Wagg C.S., Zhang L., Teoh H., Dyck J.E., Uddin G.M., Oudit G.Y., Mayoux E. (2018). Empagliflozin Increases Cardiac Energy Production in Diabetes: Novel Translational Insights Into the Heart Failure Benefits of SGLT2 Inhibitors. JACC Basic. Transl. Sci..

[20] Packer M., Anker S.D., Butler J., Filippatos G., Ferreira J.P., Pocock S.J., Sattar N., Brueckmann M., Jamal W., Cotton D. (2021). Empagliflozin in Patients with Heart Failure, Reduced Ejection Fraction, and Volume Overload: EMPEROR-Reduced Trial. J. Am. Coll. Cardiol..

[21] Hidalgo Santiago J.C., Maraver Delgado J., Cayón Blanco M., López Saez J.B., Gómez-Fernández P. (2020). Effect of dapagliflozin on arterial stiffness in patients with type 2 diabetes mellitus. Med. Clin..

[22] Klen J., Dolžan V. (2021). Treatment Response to SGLT2 Inhibitors: From Clinical Characteristics to Genetic Variations. Int. J. Mol. Sci..

[23] Verma S., McGuire D.K., Kosiborod M.N. (2020). Two Tales: One Story: EMPEROR-Reduced and DAPA-HF. Circulation.

[24] Zannad F., Ferreira J.P., Pocock S.J., Anker S.D., Butler J., Filippatos G., Brueckmann M., Ofstad A.P., Pfarr E., Jamal W. (2020). SGLT2 inhibitors in patients with heart failure with reduced ejection fraction: A meta-analysis of the EMPEROR-Reduced and DAPA-HF trials. Lancet.

[25] Anker S.D., Butler J., Usman M.S., Filippatos G., Ferreira J.P., Bocchi E., Böhm M., Rocca H.P.B., Choi D.J., Chopra V. (2022). Efficacy of empagliflozin in heart failure with preserved versus mid-range ejection fraction: A pre-specified analysis of EMPEROR-Preserved. Nat. Med..

[26] Anker S.D., Siddiqi T.J., Filippatos G., Zannad F., Ferreira J.P., Pocock S.J., Brueckmann M., Zeller C., Packer M., Butler J. (2022). Outcomes with empagliflozin in heart failure with preserved ejection fraction using DELIVER-like endpoint definitions. Eur. J. Heart Fail..

[27] Kosiborod M.N., Bhatt A.S., Claggett B.L., Vaduganathan M., Kulac I.J., Lam C.S.P., Hernandez A.F., Martinez F.A., Inzucchi S.E., Shah S.J. (2023). Effect of Dapagliflozin on Health Status in Patients with Preserved or Mildly Reduced Ejection Fraction. J. Am. Coll. Cardiol..

[28] Kusi-Yeboah T., Gianfrancesco I., Jabbar M.A.D.A., Collins P., Bally D.J., Thornton J., Williams K., Ishola A., Hong L., Toong P.J. (2025). Investigating the effect of SGLT2 inhibitors on cardiovascular related health status in HFmrEF and HFpEF: Systematic review and meta analysis. Front. Cardiovasc. Med..

[29] Bonnesen K., Heide-Jørgensen U., Christensen D.H., Lash T.L., Hennessy S., Matthews A., Pedersen L., Thomsen R.W., Schmidt M. (2024). Comparative Cardiovascular Effectiveness of Empagliflozin Versus Dapagliflozin in Adults with Treated Type 2 Diabetes: A Target Trial Emulation. Circulation.

[30] Modzelewski K.L., Pipilas A., Bosch N.A. (2024). Comparative Outcomes of Empagliflozin to Dapagliflozin in Patients with Heart Failure. JAMA Netw. Open.

[31] Lim J., Kwak S., Choi Y.J., Rhee T.M., Park C.S., Kim B., Han K.D., Lee H., Park J.B., Kim Y.J. (2024). Differing Efficacy of Dapagliflozin Versus Empagliflozin on the Risk of Incident Atrial Fibrillation in Patients with Type 2 Diabetes: A Real-World Observation Using a Nationwide, Population-Based Cohort. J. Am. Heart Assoc..

[32] Biegus J., Voors A.A., Collins S.P., Kosiborod M.N., Teerlink J.R., Angermann C.E., Tromp J., Ferreira J.P., Nassif M.E., Psotka M.A. (2023). Impact of empagliflozin on decongestion in acute heart failure: The EMPULSE trial. Eur. Heart J..

[33] Cox Z.L., Collins S.P., Aaron M., Hernandez G.A., Iii A.T.M., Davidson B.T., Fowler M., Lindsell C.J., Frank E.H., Jenkins C.A. (2021). Efficacy and safety of dapagliflozin in acute heart failure: Rationale and design of the DICTATE-AHF trial. Am. Heart J..

[34] Damman K., Beusekamp J.C., Boorsma E.M., Swart H.P., Smilde T.D.J., Elvan A., van Eck J.M., Heerspink H.J., Voors A.A. (2020). Randomized, double-blind, placebo-controlled, multicentre pilot study on the effects of empagliflozin on clinical outcomes in patients with acute decompensated heart failure (EMPA-RESPONSE-AHF). Eur. J. Heart Fail..

[35] Berg D.D., Patel S.M., Haller P.M., Bělohlávek J., Desai A.S., Drożdż J., Inzucchi S.E., McMurray J.J.V., Merkely B., O’Meara E. (2025). Rationale and Design of the Dapagliflozin Effect on Cardiovascular Events in Acute Heart Failure (DAPA ACT HF)-TIMI 68 Trial. JACC Heart Fail..

[36] Frantz S., Bauersachs J. (2022). Left ventricular remodeling after myocardial infarction. Eur. Heart J..

[37] Butler J., Jones W.S., Udell J.A., Anker S.D., Petrie M.C., Harrington J., Mattheus M., Zwiener I., Amir O., Bahit M.C. (2024). Empagliflozin after Acute Myocardial Infarction. N. Engl. J. Med..

[38] James S., Erlinge D., Storey R.F., McGuire D.K., de Belder M., Björkgren I., Johansson P.A., Langkilde A.M., Ridderstråle W., Parvaresh Rizi E. (2023). Rationale and design of the DAPA-MI trial: Dapagliflozin in patients without diabetes mellitus with acute myocardial infarction. Am. Heart J..

[39] Hernandez A.F., Udell J.A., Jones W.S., Anker S.D., Petrie M.C., Harrington J., Mattheus M., Seide S., Zwiener I., Amir O. (2024). Effect of Empagliflozin on Heart Failure Outcomes After Acute Myocardial Infarction: Insights From the EMPACT-MI Trial. Circulation.

[40] Anker S.D., Butler J., Filippatos G., Ferreira J.P., Bocchi E., Böhm M., Brunner-La Rocca H.P., Choi D.J., Chopra V., Chuquiure-Valenzuela E. (2021). Empagliflozin in Heart Failure with a Preserved Ejection Fraction. N. Engl. J. Med..

[41] Brenner B.M., Lawler E.V., Mackenzie H.S. (1996). The hyperfiltration theory: A paradigm shift in nephrology. Kidney Int..

[42] Chatur S., Vaduganathan M., Claggett B.L., Mc Causland F.R., Desai A.S., Jhund P.S., de Boer R.A., Hernandez A.F., Inzucchi S.E., Kosiborod M.N. (2023). Dapagliflozin in patients with heart failure and deterioration in renal function. J. Am. Coll. Cardiol..

[43] Coca S.G. (2023). SGLT2i and deterioration of kidney function in heart failure: Another demonstration for tolerance of “hypercreatininemia”. J. Am. Coll. Cardiol..

[44] Cox Z.L., Collins S.P., Hernandez G.A., McRae A.T., Davidson B.T., Adams K., Aaron M., Cunningham L., Jenkins C.A., Lindsell C.J. (2024). Efficacy and safety of dapagliflozin in patients with acute heart failure. J. Am. Coll. Cardiol..

[45] Schulze P.C., Bogoviku J., Westphal J., Aftanski P., Haertel F., Grund S., von Haehling S., Schumacher U., Möbius-Winkler S., Busch M. (2022). Effects of early empagliflozin initiation on diuresis and kidney function in patients with acute decompensated heart failure (EMPAG-HF). Circulation.

[46] Merlo A., D’Elia E., Di Odoardo L., Sciatti E., Senni M. (2025). SGLT2 inhibitors and new frontiers in heart failure treatment regardless of ejection fraction and setting. Eur. Heart J. Suppl..

[47] Ferreira J.P., Zannad F., Butler J., Filipattos G., Ritter I., Schüler E., Kraus B.J., Pocock S.J., Anker S.D., Packer M. (2022). Empagliflozin and serum potassium in heart failure: An analysis from EMPEROR-Pooled. Eur. Heart J..

[48] Greene S.J., Spahillari A., Shahzeb Khan M. (2024). Enabling medical therapy for heart failure: New tools bring a new reality for hyperkalemia. J. Am. Coll. Cardiol..

[49] Kittipibul V., Cox Z.L., Chesdachai S., Fiuzat M., Lindenfeld J., Mentz R.J. (2024). Genitourinary Tract Infections in Patients Taking SGLT2 Inhibitors: JACC Review Topic of the Week. J. Am. Coll. Cardiol..

[50] Puckrin R., Saltiel M.P., Reynier P., Azoulay L., Yu O.H.Y., Filion K.B. (2018). SGLT-2 inhibitors and the risk of infections: A systematic review and meta-analysis of randomized controlled trials. Acta Diabetol..

[51] Sorensen M.D., Krieger J.N., Rivara F.P., Broghammer J.A., Klein M.B., Mack C.D., Wessells H. (2009). Fournier’s Gangrene: Population based epidemiology and outcomes. J. Urol..

[52] Silverii G.A., Dicembrini I., Monami M., Mannucci E. (2020). Fournier’s gangrene and sodium-glucose co-transporter-2 inhibitors: A meta-analysis of randomized controlled trials. Diabetes Obes. Metab..

[53] Patil T., Cook M., Hobson J., Kaur A., Lee A. (2023). Evaluating the Safety of Sodium-Glucose Cotransporter-2 Inhibitors in a Nationwide Veterans Health Administration Observational Cohort Study. Am. J. Cardiol..

[54] Fralick M., Schneeweiss S., Patorno E. (2017). Risk of Diabetic Ketoacidosis after Initiation of an SGLT2 Inhibitor. N. Engl. J. Med..

[55] Meyer E.J., Gabb G., Jesudason D. (2018). SGLT2 inhibitor-associated euglycemic diabetic ketoacidosis: A South Australian clinical case series and Australian spontaneous adverse event notifications. Diabetes Care.

[56] Umapathysivam M.M., Gunton J., Stranks S.N., Jesudason D. (2024). Euglycemic Ketoacidosis in Two Patients Without Diabetes After Introduction of Sodium-Glucose Cotransporter 2 Inhibitor for Heart Failure with Reduced Ejection Fraction. Diabetes Care.

[57] Butler J., Usman M.S., Khan M.S., Greene S.J., Friede T., Vaduganathan M., Filippatos G., Coats A.J.S., Anker S.D. (2020). Efficacy and safety of SGLT2 inhibitors in heart failure: Systematic review and meta-analysis. ESC Heart Fail..

[58] Neuen B.L., Oshima M., Agarwal R., Arnott C., Cherney D.Z., Edwards R., Langkilde A.M., Mahaffey K.W., McGuire D.K., Neal B. (2022). Sodium-Glucose Cotransporter 2 Inhibitors and Risk of Hyperkalemia in People with Type 2 Diabetes: A Meta-Analysis of Individual Participant Data From Randomized, Controlled Trials. Circulation.

[59] Morace C., Lorello G., Bellone F., Quartarone C., Ruggeri D., Giandalia A., Mandraffino G., Minutoli L., Squadrito G., Russo G.T. (2024). Ketoacidosis and SGLT2 Inhibitors: A Narrative Review. Metabolites.

[60] Azmi Y.A., Alkaff F.F., Soetanto K.M., Wirjopranoto S., Postma M.J., Purba A.K.R. (2025). The impact of sodium-glucose cotransporter-2 inhibitors on the incidence, therapy, and outcomes of fournier gangrene: Insights from a systematic review of case reports. Syst. Rev..

[61] Chen H.B., Yang Y.L., Meng R.S., Liu X.W. (2023). Indirect comparison of SGLT2 inhibitors in patients with established heart failure: Evidence based on Bayesian methods. ESC Heart Fail..

[62] Davis J.A., Booth D., McEwan P., Solomon S.D., McMurray J.J.V., de Boer R.A., Comin-Colet J., Bachus E., Chen J. (2024). Cost-effectiveness of dapagliflozin for patients with heart failure across the spectrum of ejection fraction: A pooled analysis of DAPA-HF and DELIVER data. Eur. J. Heart Fail..

[63] Reifsnider O.S., Tafazzoli A., Linden S., Ishak J., Rakonczai P., Stargardter M., Kuti E. (2024). Cost-Effectiveness Analysis of Empagliflozin for Treatment of Patients with Heart Failure with Reduced Ejection Fraction in the United States. J. Am. Heart Assoc..

[64] Rao V.N., Murray E., Butler J., Cooper L.B., Cox Z.L., Fiuzat M., Green J.B., Lindenfeld J., McGuire D.K., Nassif M.E. (2021). In-Hospital Initiation of Sodium-Glucose Cotransporter-2 Inhibitors for Heart Failure with Reduced Ejection Fraction. J. Am. Coll. Cardiol..

[65] Stolfo D., Lund L.H., Benson L., Lindberg F., Ferrannini G., Dahlström U., Sinagra G., Rosano G.M.C., Savarese G. (2023). Real-world use of sodium-glucose cotransporter 2 inhibitors in patients with heart failure and reduced ejection fraction: Data from the Swedish Heart Failure Registry. Eur. J. Heart Fail..

[66] Rashed A., Wasef M., Kalra P.R. (2024). The 2023 ESC heart failure guideline update and its implications for clinical practice. Br. J. Cardiol..

[67] Isaza N., Calvachi P., Raber I., Liu C.L., Bellows B.K., Hernandez I., Shen C., Gavin M.C., Garan A.R., Kazi D.S. (2021). Cost-effectiveness of Dapagliflozin for the Treatment of Heart Failure with Reduced Ejection Fraction. JAMA Netw. Open.

[68] Ferreira J.P., Vasques-Nóvoa F., Saraiva F., Oliveira A.C., Almeida J., Batista A.B., Barbosa A., Ferreira A.F., Costa C., Diaz S.O. (2025). Sodium-Glucose Cotransporter 2 Inhibitor with and Without an Aldosterone Antagonist for Heart Failure with Preserved Ejection Fraction: The SOGALDI-PEF Trial. J. Am. Coll. Cardiol..

[69] Liu T., Fan Z., Li Y., Xiao B., He C. (2025). Combination treatment of SGLT2i and GLP-1RA associated with improved cardiovascular outcomes in type 2 diabetes patients with acute coronary syndrome: A propensity score-matched cohort study. Int. J. Cardiol..

[70] A Study to Determine the Efficacy and Safety of Finerenone and SGLT2i in Combination in Hospitalized Patients with Heart Failure (CONFIRMATION-HF) (CONFIRMATION), ClinicalTrials.gov ID NCT06024746. NCT06024746.

[71] Phase III Study Investigating Heart Failure and Cardiovascular Death with Baxdrostat in Combination with Dapagliflozin (Prevent-HF), ClinicalTrials.gov ID NCT06677060. NCT06677060.

[72] Ezhumalai B., Modi R., Panchanatham M., Kaliyamoorthy D. (2024). The contemporary role of sodium-glucose co-transporter 2 inhibitor (SGLT2i) and angiotensin receptor-neprilysin inhibitor (ARNI) in the management of heart failure: State-of-the-art review. Indian Heart J..

